# GLA:D® Back: group-based patient education integrated with exercises to support self-management of persistent back pain — feasibility of implementing standardised care by a course for clinicians

**DOI:** 10.1186/s40814-019-0448-z

**Published:** 2019-05-09

**Authors:** Alice Kongsted, Jan Hartvigsen, Eleanor Boyle, Inge Ris, Per Kjaer, Line Thomassen, Werner Vach

**Affiliations:** 1 Department of Sports Science and Clinical Biomechanics, University of Southern Denmark, Odense, Denmark; 2 Nordic Institute of Chiropractic and Clinical Biomechanics, Odense, Denmark; 3 Health Sciences Research Centre, University College Lillebaelt, Odense, Denmark; 4 Department of Orthopaedics and Traumatology, University Hospital Basel, Basel, Switzerland

**Keywords:** Back pain, Implementation, Patient education, Exercise, Self-management, Pilot study, Feasibility

## Abstract

**Background:**

Clinical guidelines for management of low back pain (LBP) are not routinely implemented in practice, and guidelines rarely offer tools for implementation. Therefore, we developed GLA:D® Back, a standardised intervention of patient education and supervised exercises. This pilot study tested the feasibility of implementing GLA:D Back in clinical practice in Denmark by delivering a course for physiotherapists and chiropractors. It should further inform the planning of an implementation-effectiveness study using a pre-post group design alongside nation-wide implementation.

**Methods:**

Thirty-one clinicians from nine clinics participated. Feasibility of implementation was evaluated in terms of adoption and through focus group interviews and a feedback meeting. Patient-level data, including pain, disability, and pain enablement, were collected from (1) LBP patients visiting the clinics during a pre-specified 2-week period 2 months prior to clinicians attending the GLA:D Back course (*n* = 84), (2) LBP patients consulting during a 2-week period 2 months after the course (*n* = 77), and (3) those enrolled in GLA:D Back during 4 months after implementation (*n* = 89). Patient data were collected at baseline and at 4 months.

**Results:**

Clinicians’ evaluations of the course were positive and resulted in several modifications. The clinical intervention was adopted by all test sites. Most patient characteristics were similar across groups. Patients mainly had persistent LBP (73% > 3 months) and most had been treated for more than 4 weeks at inclusion. Patients in GLA:D Back were more often retired (30% vs. 16% before implementation) and at high risk of poor prognosis (25% vs. 13%). Procedures for data collection were feasible, and outcomes after implementation, especially with GLA:D Back, were as good as or better than before implementation. Recruiting patients and achieving comparable pre- and post-groups was difficult.

**Conclusions:**

Implementation of the GLA:D Back clinical intervention in Danish primary care physiotherapy and chiropractic clinics was feasible through a 2-day clinician course. Both clinicians and patients were satisfied with the programme, and patient-reported outcomes were slightly better than outcomes in patients registered before implementation. It was not deemed possible to conduct an implementation-effectiveness trial as part of a nation-wide implementation.

**Electronic supplementary material:**

The online version of this article (10.1186/s40814-019-0448-z) contains supplementary material, which is available to authorized users.

## Background

Clinical guidelines for the treatment of back pain consistently recommend that patients are educated about their condition and are encouraged to remain physically active and at work while some include supervised exercise therapy that may or may not be combined with manual therapies [1]. However, these recommendations are not routinely implemented in clinical practice [2] causing many people to receive care that is ineffective [3, 4].

Standardised care packages can facilitate the translation of guideline recommendations into clinical practice [5, 6]. *Good Life with osteoArthritis in Denmark* (GLA:D®) for patients with knee or hip pain is one programme that has succeeded in making a standardised evidence-based care package widely available [5]. From 2013 to 2017, more than 1100 clinicians were trained in that programme in Denmark alone, and over 30,000 patients have entered a clinical registry used for monitoring outcomes [5, 7]. Education of clinicians in delivering physical and educational interventions for people seeking care for back pain has also been used in effectiveness trials [6, 8, 9]. Similarly, GLA:D Back is a standardised package developed to assist clinicians in delivering evidence-based patient education and exercise therapy for people seeking care for persistent or recurrent back pain. It is taught to clinicians in a 2-day course and monitored in a clinical registry [10].

The GLA:D Back care package is based on interventions shown effective in clinical trials, and initial testing at our university clinic suggested that it was both feasible and perceived worthwhile by patients. However, inference of effects found in randomised clinical trials into everyday clinical practice is uncertain, and effective implementation strategies within back pain have not yet been established [11, 12]. Thus, it is yet unknown if the successful implementation of the GLA:D approach used for knee and hip pain at the national level will also work for back pain.

This pilot study was conducted to test the feasibility of implementing GLA:D Back in community-based physiotherapy and chiropractic clinics in order to prepare for a nationwide implementation of GLA:D Back paired with an implementation-effectiveness trial. A stepped-wedge design with geographical regions as clusters would be a natural choice for testing effectiveness, as it allows spreading the training of clinicians over a longer period and it allows inclusion of the entire national patient population. Such a design requires recruiting, one region at a time, one patient group before offering training to clinicians in that same region and one patient group after the training of clinicians. With this design, we will be able to evaluate effects at two levels of intervention, namely an implementation intervention consisting in training courses for clinicians, and a clinical intervention, which is the GLA:D Back patient education and exercises delivered by trained clinicians. The overall effects of the implementation would then be the combined effects of patients having a potentially more effective clinical intervention than the pre-existing usual care, as well as effects of shifts in the patient population that is offered this type of care. Cost-effective recruitment of patients can be undertaken by the participating clinicians, and a lack of a central registry covering the relevant patients makes alternative approaches cumbersome. Hence, we will focus on the aspects of patient recruitment and comparability of pre- and post-patient groups. Furthermore, the pilot study should inform us about data collection procedures which are part of the planned GLA:D Back registry, covering all patients enrolled in the programme. In summary, the objectives of this study were as follows:

1. To explore participating clinicians’ perceptions of the training course and the GLA:D Back intervention
2. To explore the adoption of GLA:D Back in the clinics
3. To test the administration of questionnaires for determining clinicians’ confidence and back beliefs and the potential for capturing any change on these scales
4. To describe the characteristics of the patients enrolled in the GLA:D Back programme in order to determine who clinicians consider to be candidates for GLA:D Back
5. To estimate changes in patient outcomes with the care provided before and after implementation (usual care) and with GLA:D Back
6. To test the data collection procedures planned as part of the GLA:D Back registry
7. To test the recruitment of patients for pre- and post-group by participating clinicians
8. To evaluate the usefulness of outcome measures in terms of completeness of responses, and
9. To identify areas of the GLA:D Back programme that need to be modified

## Methods

### Setting and design

The study was conducted at the University of Southern Denmark and in community-based physiotherapy and chiropractic clinics in Denmark. Clinicians participated in a training course at the university in delivering the GLA:D Back intervention, and subsequently, they delivered the clinical intervention to their patients at their clinics. Clinicians and patients responded to electronic questionnaires in Research Electronic Data Capture (REDCap) that is licenced by the Open Patient data Explorative Network (OPEN).

**Fig. 1:**
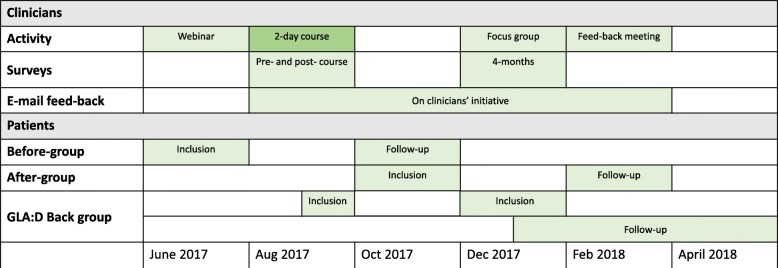
Study flow. Rough overview of activities and data collections at the level of clinicians and patients. Please refer to the text for exact time periods

At the clinician level, the study was a longitudinal cohort study with clinician completed questionnaires on back pain beliefs before the course and 4 months later (Fig. 1).

At the patient level, we compared three groups of patients and each group was followed for 4 months. Patients in the first group sought care from the clinicians prior to the clinicians being trained in the GLA:D Back programme (*before-group*), and the second group after the clinicians had participated in the training (*after-group*). Both of these received treatment at the discretion of the clinician, with the GLA:D Back clinical intervention as one of the options after its implementation. In addition, we included all patients enrolled in GLA:D Back up until 4 months after the clinician course (*GLA:D Back group*) in order to recruit a volume of GLA:D Back participants that would allow determining the characteristics of the target group for GLA:D Back and estimating their outcomes.

The implementation process was evaluated using a mixed-method design. A sample of participating clinicians took part in a focus group interview, at least one clinician from each clinic participated in a feedback meeting, and additional feedback was received by mail on the clinicians’ own initiative during the study period. Evaluation of the adoption of the intervention and the clinicians’ quantitative feedback on the clinical intervention was based on patient registrations in the clinical registry and clinician-completed questionnaires at the 4-month follow-up.

### Study participants

#### Clinicians

Invitations to participate in this study were sent to five physiotherapy clinics and four chiropractic clinics who had expressed interest in participating after having heard about the development of GLA:D Back or had been involved in previous research projects with our group. All clinics accepted their invitation. We chose this group of clinicians to explore if implementation of the GLA:D Back intervention would be feasible with highly motivated clinicians since otherwise the strategy would need to be substantially changed. The selection ensured that both clinics with and without prior experience with GLA:D knee/hip were represented. It was up to the clinics to decide who and how many of their clinicians would participate in the 2-day GLA:D Back course. All participating clinicians provided their consent for the data to be used for research purposes.

#### Patients

The clinicians at each clinic recruited three groups of patients. The *before-group*, recruited prior to clinicians being trained in GLA:D Back implementation, sought care between June 19 and July 7, 2017; the *after-group* sought care between October 23 and November 30, 2017, which was after the clinicians had been trained; and the *GLA:D Back group* were patients enrolled in the GLA:D back programme between August 23, 2017, when clinicians finished the course and December 31, 2017, except for those enrolled during recruitment of the after-group.

In order to enrol in the study, patients attended one of the clinics for non-specific low back pain (LBP), were aged 18 years or older, and could speak and read Danish.

For both the before- and after-groups, patients were required either to have experienced LBP affecting daily activities for at least 1 month or to have experienced three or more episodes of LBP within the past 2 years for which they had sought care. Patients with signs of nerve root involvement or patients who were already in a supervised exercise programme were not included.

There were no firm inclusion and exclusion criteria for the GLA:D Back group. Clinicians decided in collaboration with the patients whether the intervention would be suitable for them reflecting daily practice. At the training course, clinicians were informed that the intervention was designed for patients with persistent or recurrent non-specific LBP that limits daily activities.

The first patient-completed questionnaire contained information about the study’s purpose and their rights as a study participant as well as consent to their data being used for research purposes.

### Training of clinicians

Clinicians first participated in a 1-h webinar in June 2017 that explained the objectives of the pilot study, the inclusion criteria for the before- and after-groups, the procedures for the clinical tests (described under “patient data”) that were being used and a demonstration of the web-based data capturing tool. The webinar was recorded and made available to the clinicians afterwards.

On August 22 to 23, 2017, they participated in a 2-day course at the University of Southern Denmark (Fig. 1). The course was a mixture of lectures and practical workshops [13] and aimed to develop the clinicians’ ability to deliver the GLA:D Back programme (described in the “The GLA:D Back intervention” section below) by introducing all its elements: goal setting, clinical tests, patient education, supervised exercises, and data registrations. Role-playing and skills training were used to become familiar with pain education, physical tests, and exercises. Using some of the slides from the patient education material, participants worked in groups with ways of delivering key messages from the pain education as they would in a real patient session. Clinical tests and exercises were introduced in a practical session with tests carried out on a colleague and exercises performed by the participants. After the course, participants were given access to a closed website that contained materials for patient education sessions (PowerPoint with manuscript, exercises to support patients’ reflections, two posters with patient education key messages), exercise programmes, and information folders directed at patients and primary care physicians about the pilot project.

### Interventions

#### Interventions in the before- and after-group

No restrictions were put on the interventions for patients in the before- and after-groups. They were offered the types of care that clinicians found appropriate. For patients in the after-group, however, the GLA:D Back intervention was a treatment option. Patients in the before-group who were in active treatment during the GLA:D back recruitment phase could be enrolled in GLA:D Back before their 4-month follow-up.

#### The GLA:D Back intervention

The overall aim of GLA:D Back is to support patients’ self-efficacy and self-management by providing them with an understanding of pain mechanisms, reducing their fear of movement, and supporting patients in gaining control of pain and in moving freely.

The GLA:D Back programme has been described in detail elsewhere and is only briefly outlined here [10]. The programme starts with an individual session where personal goals are discussed, clinical tests performed, and the starting level for exercises tested. This is followed by the clinical intervention consisting of two 1-h patient education group sessions and bi-weekly 1-h supervised exercise sessions for 8 weeks. A group size of 6–8 patients was recommended, and the maximum group size allowed was a group of 10. The programme ends with a final individual session where personal goals are revised and clinical tests repeated.

The intervention was developed around the social cognitive theory and the cognitive behavioural theory. Education and movement are the tools used to support the development of self-efficacy. The key messages (for example, back pain is common, pain intensity does not reflect tissue injury, and the spine is strong and designed for movement) are stressed throughout the patient education sessions and integrated with the supervised exercise sessions. Further to this, the patients’ existing beliefs and concerns are addressed.

The GLA:D Back exercise programme includes strength, endurance, and flexibility training. Each exercise has four levels of difficulty, and each patient starts at the level that is deemed suitable for her or him. During the exercises, patients are encouraged by their clinician to explore varieties of movement rather than doing exercises in one “correct” manner. Patients are also encouraged to make decisions about progression of exercise difficulty, while clinicians guide the performance of exercises and the choice of exercise level to the degree needed. The exercise programme is thus individualised within a standardised frame.

### Methods and types of data collections

#### Clinician data

Clinicians contributed survey information at three time-points: 1 week prior to participating in the GLA:D Back course, immediately after the GLA:D Back course, and 4 months after the GLA:D back course.

One week prior to the GLA:D Back course, clinicians received a link to an electronic survey that included questions about their characteristics (age, sex, profession, years of clinical experience etc.), their confidence (Practitioner Confidence Scale—PCS), and attitudes and beliefs (the Pain Attitudes and Beliefs Scale—PABS) about back pain (Table 1). The PCS and PABS were repeated 4 months after the course.

**Table 1:** Clinician reported measures and time-points for data collection

Construct	Instrument	Before course	After course	4 months	Patient baseline registration
Individual characteristics	Age, sex, profession, role in the clinic	x			
Experience	Years of clinical experience	x			
	Experience with GLA:D for knee and hip				
Confidence	The Practitioner Confidence Scale (range 4–20) [14]	x		x	
Treatment orientation	The Pain Attitudes and Beliefs Scale for Physiotherapists [15–17]. Biomedical subscale (range 10–60), behavioural subscale (range 9–54)	x		x	
Evaluation of 2-day course	Content (0 = very poor; 10 = excellent)		x		
	Novelty (0 = very low; 10 = very high)				
	Usability (0 = very poor; 10 = very good)				
Adoption	Did you start offering GLA:D Back in the clinic?			x	x
	How many groups have started?				
	Number of patients in the registry				
Overall impression	Everything considered, how do you think GLA:D Back works? (5-points Likert: very bad to very good)			x	
Materials	How satisfied are you with the patient education materials? (0 = very unsatisfied; 10 = very satisfied)			x	
	How satisfied are you with the exercise programme? (0 = very unsatisfied; 10 = very satisfied)				

The PCS is a 4-item scale measuring confidence with managing people with back pain [14]. Each item is scored on a 5-point scale from 1 = “strongly agree” to 5 = “strongly disagree”, resulting in sum scores ranging from 4 to 20 where a higher score indicates a lower confidence.

The PABS was developed to assess the strength of two possible treatment orientations of physiotherapists toward the management of back pain: predominantly biomedical orientation or predominantly behavioural orientation [15, 16]. The biomedical subscale consists of 10 items (sum score 10 to 60) and the behavioural subscale of 9 items (sum score 9 to 54). Higher scores reflect a more biomedical or behavioural orientation respectively.

The 4-month questionnaire also asked about the clinicians’ experience with GLA:D Back and their satisfaction with the patient education materials and exercise programme (Table 1). In addition to these outcomes, data on implementation was collected using the Determinants of Implementation Behaviour Questionnaire [18], which was developed to evaluate domains influencing implementation. These data are reported separately (Ris I, Schröder K, Kongsted A, Abbott A, Nilsen P, Hartvigsen J, et al. Modification of the Determinants of Implementation Behaviour Questionnaire (DIBQ) to evaluate practitioner use of best practice primary health care programs for low back pain in Sweden and Denmark. In preparation).

In a questionnaire sent immediately after the course, the clinicians rated the course in respect of course content, novelty, and usefulness on a 0 to 10 NRS scale for each aspect (from 0 = “very poor” to 10 = “very good”).

Four months after the clinicians’ course, a focus group interview was conducted at the university by a research assistant with participation from four clinics. Participants were purposively selected based on whether they reported either the least or the most challenges with implementing GLA:D Back as measured by the Determinants of Implementation Behaviour Questionnaire [18]. The interview guide was centred on perspectives on the content of the clinical intervention and the implementation at their clinic, with perspectives on recruitment for the before- and after-groups as a secondary topic. The focus group was audio recorded, and quotes related to the 2-day course, the patient education, the exercise therapy, and data registration into REDCap were identified independently by two researchers (IR and AK) and translated to English for the reporting of results.

In February 2018, all clinics were invited to participate in a feedback meeting about the training course, their experiences with the GLA:D Back programme, and their thoughts on its strengths, weaknesses, opportunities, and threats.

During the whole study period, clinicians provided informal feedback via email on any aspect they found needed attention.

#### Patient data

##### Self-reported data

Patients who accepted to be part of the study had their e-mail address registered by the clinician and received an automatically generated link to a survey on the day of the baseline consultation and 4 months later. If there was no response within 3 days, an automated reminder was sent.

The baseline questionnaire collected demographic information, information on LBP history and previous treatment, and self-reported risk factors for a poor prognosis (The START Back Screening Tool) (Table 2).

**Table 2:** Patient outcome measurements

Construct	Instrument	Baseline	4 months	Clinician-reported pre-intervention	Clinician-reported post-intervention
Demographics	Gender, age	x			
	Education (no qualification, vocational training, higher education < 3 years, higher education ≥ 3 years)				
Work situation	Job type (ordinary work, unemployed, rehabilitation, retired, student/housewife/other)	x			
LBP history	Pain duration (< 4 weeks, 4–12 weeks, 3–12 months, or > 1 year)	x			
	Previous number of episodes of back pain (0, 1, 2–3, or > 3)				
	Time since treatment was initiated for the current episode (< 2 weeks, 2–4 weeks, or > 4 weeks)				
	Number of health care visits for the current episode (1, 2–5, 6–10, or > 10)				
Risk profile	The START Back screening tool (low risk, medium risk, high risk of poor prognosis) [19]	x			
Activity limitation	Oswestry Disability Index (0–100; higher scores reflect more disability) [20, 21]	x	x		
Pain intensity	Numeric Rating Scale 0–10 for LBP and leg pain (0 = no pain, 10 = worst imaginable pain) [22]	x	x		
Sick leave	Number of days of sick leave in the last 3 months in those who were working (0 days, 1–14 days, or > 14days)	x			
Illness perceptions	The Brief Illness Perceptions Questionnaire [23, 24] (range 0–80; higher scores reflect more threatening view)	x	x		
Fear of movement	Fear Avoidance Beliefs Questionnaire, subscale: physical activity (0–24; higher scores reflect more fear-avoidance beliefs) [25, 26]	x	x		
Quality of life	Mental wellbeing: SF-36 subscale transformed (0–100; 0 = very poor, 100 = very good)	x	x		
	Social functioning limited by physical health: SF-36 item 6. 1 = not at all, 5 = extremely. Impaired social function defined as scores 3 (moderate) to 5 (extremely)				
Self-efficacy	The Back Pain Enablement Instrument (range 0–60; higher scores reflect higher sense of enablement). Modified from the Pain Enablement Instrument [27, 28]	x	x		
Perceived physical fitness	Sum score of self-assessed strength, endurance, cardiovascular fitness, balance (range 0–40; higher scores reflect better perceived fitness) [29]	x	x		
Pain medication	Current use of over the counter or prescribed pain medication (yes/no)	x	x		
Physical back performance	Standing forward bending (0: did not perform; 1: performed with pain and with unusual movement; 2: performed without pain and with unusual movement; 3: performed with pain and with normal movement; 4: Performed without pain and with normal movement) [30]			x	x
	Trunk flexor endurance test (seconds in static flexion) [31, 32]				
	The Ito back extensor endurance test (seconds in static extension) [31, 33]				
	Active straight leg raise (0: no leg lift; 1: pain with leg lifting not disappearing with activation of abdominals; 2: pain with leg lifting that disappear with activation of abdominals; 3: no pain with leg lifting (0–2 = impaired)) [34]				
Content of intervention	Received listed intervention (yes/no)		x		
	Advice about daily activities				
	Thorough information (education individually or in groups)				
	Exercise therapy (individually or in groups)				
	Manual therapy				
	Massage				
	Passive modalities or needles (e.g. laser, ultrasound, acupuncture)				
Satisfaction	Overall, are you satisfied with your course of care (5-point Likert)		x		

Both at baseline and the 4-month follow-up, patients were asked about pain intensity (0–10 NRS), pain medication for LBP (yes/no), activity limitation (Oswestry Disability Index), illness perceptions (the Brief Illness Perceptions Questionnaire—B-IPQ), fear of movement (Fear Avoidance Beliefs Questionnaire—FABQ), quality of life (SF-36 subscales mental wellbeing and social functioning limited by physical health), self-efficacy (the Back Pain Enablement Instrument—BPEI), and perceived physical fitness (self-assessed strength, endurance, cardiovascular fitness, balance) (Table 2).

In addition, the 4-month questionnaire contained questions whether the participant had received any of the eight listed interventions (individual patient education/thorough information, group-based patient education/thorough information, individual exercise therapy, group-based exercise therapy, manual therapy, massage, passive modalities including acupuncture, laser, ultrasound) or other treatments since their baseline visit. They were also asked about their satisfaction with care.

##### Clinician reported data

Participating clinicians performed a series of clinical tests on their patients during the scheduled individual sessions. Physical performance was tested as standing forward bending [30, 35, 36], the Ito extensor endurance test (seconds in static extended position up till 3 min) [31, 33], the trunk flexor endurance test (seconds in static sit-up position up till 2 min) [31, 32]), and the active straight leg test (ASLR) (0: no leg lift; 1: pain with leg lifting not disappearing with activation of abdominals; 2: pain with leg lifting that disappear with activation of abdominals; 3: no pain with leg lifting (0–2 = impaired)) [34].

### Sample size

In each of the 2 sampling periods before and after the implementation, it was expected that 10 patients would be included per clinician resulting in two groups of 300 patients. In the GLA:D Back group, we expected at least 54 patients corresponding to each clinic initiating 1 group of 6 patients. No formal sample size calculation was performed.

### Analyses

The clinician course was evaluated by describing medians and the range of scores on the items evaluating the course. Clinicians’ qualitative feedback was pragmatically summarised by grouping quotes thematically from the focus group interview, the feedback meeting, and emails into themes relating to the clinician course, the data registration, the patient education, and the exercises.

Adoption was evaluated based on the number of clinics that offered the GLA:D Back programme within the study period and the number of patients enrolled in GLA:D Back.

Clinicians’ back beliefs were evaluated by describing group medians, 25th and 75th percentiles on the PCS, and the PABS at baseline and 4-month follow-up. To judge the within-clinician change on the PABS, the mean change scores were calculated with standard deviations (SD).

Patient profiles were described as distributions on baseline parameters in the three patient groups, and outcomes as observed mean change from baseline to follow-up with a 95% confidence interval and as change after adjustment for baseline differences. The adjusted change scores were estimated in hierarchical models taking clustering effects of clinics into account and reported as marginal means. For binary outcomes, we used a hierarchical logit model and reported marginal proportions.

The feasibility of procedures for conducting a full-scale implementation-effectiveness trial was determined based on recruitment and follow-up rates, on whether the before and after comparison groups were sufficiently comparable, and on the extent to which outcome measures were considered feasible in the target group.

Data analyses were performed in STATA/MP15.1 (StataCorp LLC, TX, USA).

## Results

### Participating clinicians and course evaluations

Thirty-one clinicians (25 physiotherapists, 6 chiropractors) participated in the course, with overall varying clinical experience and some with previous experience with GLA:D for knee and hip pain (Table 3). One clinician did not complete the baseline questionnaire, and 2 clinicians did not respond to the 4-month questionnaire. Each clinic was represented by at least 3 clinicians. Seven out of 9 clinics were offering GLA:D for knee and hip pain.

**Table 3:** Clinician characteristics and outcomes

	*n* (%) (unless other specified)
Age, mean (range)	41 (26–58)
Female	17 (55%)
Physiotherapist	25 (81%)
Chiropractor	6 (19%)
Clinic owner	9 (30%)
Self-employed in a clinic own by someone else	11 (37%)
Employee	10 (33%)
Clinical experience	
0–5 years	9 (29%)
6–10 years	7 (23%)
11–20 years	9 (29%)
> 20 years	6 (19%)
Previous experience with GLA:D for knee/hip	
No experience	4 (14%)
Have referred to GLA:D in house	16 (55%)
Have referred to GLA:D in another clinic	3 (10%)
Have instructed GLA:D groups	6 (21%)
Evaluation of the course, median (range)	
Content (0–10)	9 (6–10)
Usability (0–10)	9 (6–10)
Novelty (0–10)	7 (2–10)
Overall impression of the GLA:D Back programme	
Very good	11 (38%)
Good	14 (48%)
Neither good nor bad	4 (14%)
Bad	0
Very bad	0
Satisfaction with patient education materials^#^	
Very satisfied	6 (32%)
Satisfied	11 (58%)
Neither satisfied nor dissatisfied	2 (10%)
Dissatisfied	0
Very dissatisfied	0
Satisfaction with the exercise programme ^#^	
Very satisfied	4 (21%)
Satisfied	12 (63%)
Neither satisfied nor dissatisfied	1 (5%)
Dissatisfied	2 (11%)
Very dissatisfied	0
Practitioner Confidence Scale (4–20), median (IQR)	
Before course	16 (13–17)
At 4 months	16 (15–18)
PABS biomedical before course (10–60), median (IQR)	30 (29–36)
PABS biomedical at 4 months (10–60), median (IQR)	27 (23–32)
PABS behavioural before course (9–54), median (IQR)	39 (36–43)
PABS behavioural at 4 months (9–54), median (IQR)	41 (38–45)

*PABS* Pain Attitudes and Beliefs Scale

^#^Only clinicians who have delivered the clinical intervention (*n* = 19)

The evaluation of the clinician course provided high scores for course content and usability and slightly lower for novelty (Table 3). This was supported by the group interview and the feedback meeting by statements such as:

overall very good materials, nice to have some repetition of things [things that were known to the participants but partly forgotten], good analogies that I have taken to heart.

The need to describe what are the core elements of the GLA:D Back clinical intervention and what elements could be modified to suit their patients and practise style was stressed in the clinicians’ feedback as exemplified by:

... your [the research team’s] guidance has to be on how you can recognise that it is GLA:D Back [that is going on] if you visit us.

### Adoption of GLA:D Back

All clinics offered GLA:D Back to their patients within the study period. From 1 to 4 groups were initiated per clinic, with 4 to 18 patients enrolled in the programme per site. The clinical intervention was delivered by 19 clinicians (14 delivering the full intervention, 3 patient education only, 2 exercise therapy only). Six of the 10 clinicians who did not deliver the intervention were clinic owners who participated in the course because they wanted to be familiar with the content of GLA:D Back and not with an intention of delivering it personally.

### Clinician feedback from 4-month survey and focus group

Clinicians’ overall impression of the programme was positive, and generally, they were satisfied with the educational materials and exercise programme although two were not satisfied with the exercises (Table 3).

In the focus group, the importance of patient education was emphasized:

I think the theory is at least as important as the exercises,

said that they [the patients] used it a lot during the course…thought about what we had said,

they [the patients] are not as afraid anymore when they have pain,

I think it really makes sense to have them [the patients] in such a forum with patient education… provides option for dialogue … they came forward with their stories

In relation to the exercise programme, much of the feedback concerned uncertainty regarding whether the exercises could be adapted, for example:

do we have to strictly work through the exact exercises or is it just e.g. abdominal training that can be adapted to the individual patient?,

we did free-style a little

Some clinicians found that the highest level of exercises was not sufficiently demanding:

to some patients it simply isn’t difficult enough

and some expressed a need for less demanding options:

…had to adjust exercises a lot for those [with severe pain] to be able to participate and benefit from it

### Input from clinician feedback meeting and emails

The same themes as revealed during the focus group interview were identified from other sources of feedback as well. In addition, it appeared that clinicians were uncertain about the rationale for the approach to exercises. For example:

what is the intention with exercises for flexibility? … that needs to be articulated,

to me the number of repetitions [performed of each exercise] seemed culturally determined more than a conscious choice,

it was not until I had been in a 3-day workshop about cognitive functional therapy that I got the messages from the patient education [referring to the GLA:D Back materials] sorted out, and how to implement them in the training sessions [referring to the GLA:D Back exercise training programme]

### Clinician outcomes

**Fig. 2:**
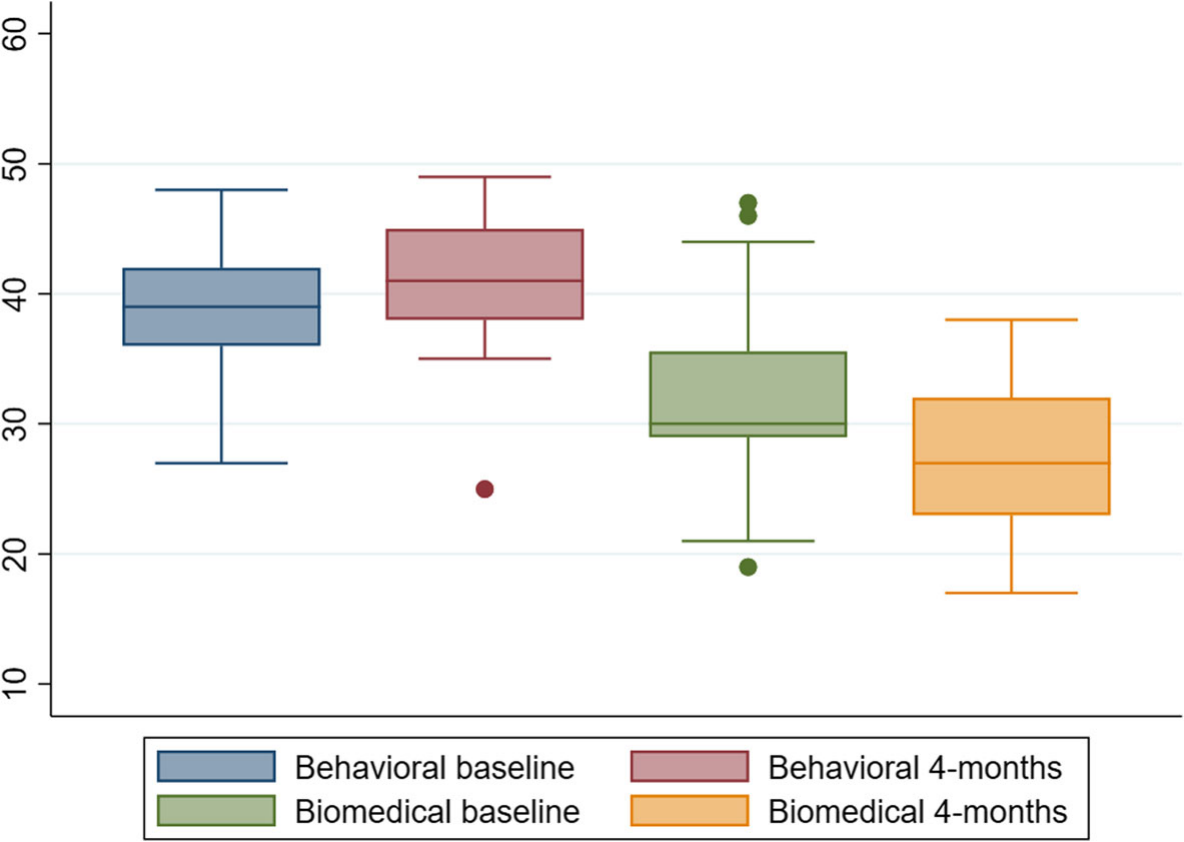
Clinicians’ scores on two subscales of the Pain Attitudes and Beliefs Scale before course participation and 4 months later

Scores on the PCS were generally high and unchanged at a group level from before the course to follow-up (Table 3). The PABS indicated that clinicians generally had a combined behavioural and biomedical orientation with some preference for the behavioural (Fig. 2). A small average change was observed over time towards a less biomedical (mean change − 4.2, SD = 6.4) and more behavioural orientation (mean change 2.1, SD = 4.4) from before the course till 4 months later (Table 3 and Fig. 2). As indicated by the standard deviations, substantial changes were observed for some clinicians.

### Patient recruitment and characteristics

The 9 clinics recruited a total of 250 patients (range 19 to 51). Eighty-four patients were enrolled in the before-group and 77 patients in the after-group. From these 77 patients, 8 patients (10%) were enrolled in GLA:D Back.

Between August 30 (1 week after the course) and December 15, 2017, additional 89 patients were enrolled in GLA:D Back outside of the recruitment period for the after-group. Five patients were excluded from the analyses because they were not enrolled in GLA:D Back and their enrolment occurred after the clinician course and outside of the enrolment time-frame for the after-group. This indicated that the procedures were either misunderstood or data from these patients were entered incorrectly into the database.

**Fig. 3:**
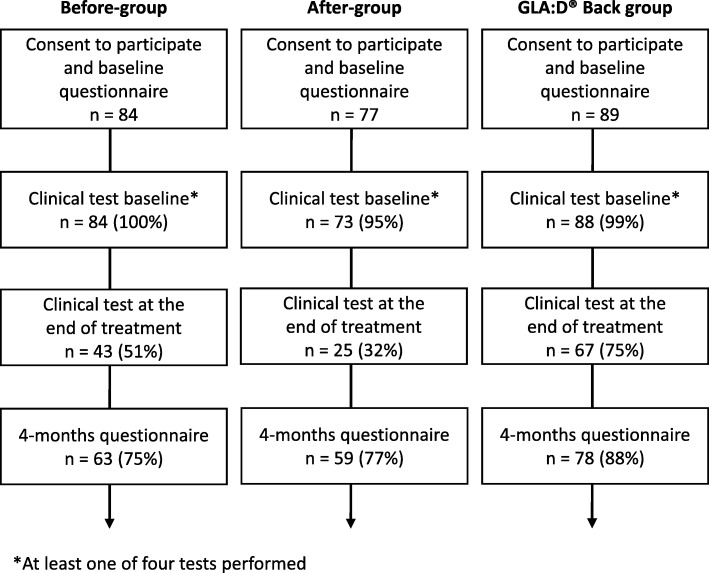
Patient flow chart. Before-group = participants recruited prior to implementation of GLA:D Back; after-group = participants recruited after the implementation

Across groups, 200 (80%) patients responded to the 4-month follow-up (Fig. 3). Response rates at 4-month follow-up were 75%, 77%, and 88% in the before-group, the after-group, and for GLA:D Back participants respectively. The clinical tests at the end of treatment were often not performed with completion rates of 51%, 32%, and 75% in the three groups respectively.

The three groups were comparable on most parameters, and included patients were mainly working, had experienced LBP for more than 3 months, received treatment for more than 4 weeks, had moderate pain and disability levels, and had B-IPQ scores reflecting that LBP was perceived as medium threatening (Table 4). Still, the before-group included fewer patients who had back-related sick leave and a smaller proportion with social impairments than the groups recruited after course participation. The after-group included a smaller proportion that reported long episode duration or had received treatment for a long period as compared to the other groups, and patients enrolled in the GLA:D Back group were on average older and more often retired (Table 4).

**Table 4:** Patient reported baseline characteristics

	Missing values, % of responders to baseline questionnaire (*n* = 250)	Before-group (*n* = 84)	After-group (*n* = 77)	GLA:D Back group (*n* = 89)
Females, *n* (%)	1%	50 (60%)	49 (65%)	57 (65%)
Age, mean (SD)	0.4%	47.8 (13.7)	46.4 (15.5)	54.8 (13.4)
No qualification	6%	12 (16%)	13 (17%)	10 (12%)
Vocational training		22 (29%)	25 (33%)	20 (24%)
Higher education < 3 years		11 (15%)	12 (16%)	12 (14%)
Higher education > 3 years		30 (40%)	25 (33%)	42 (50%)
Ordinary work	2%	53 (65%)	47 (62%)	52 (59%)
Unemployed		4 (5%)	4 (5%)	0 (0%)
Rehabilitation		3 (4%)	5 (7%)	4 (5%)
Retired		13(16%)	11 (14%)	31 (30%)
Student/housewife/other		9 (11%)	9 (12%)	5 (6%)
Pain duration	1%			
< 4 weeks		10 (12%)	15 (20%)	7 (8%)
4–12 weeks		11 (13%)	14 (18%)	8 (9%)
3–12 months		21 (25%)	19 (25%)	28 (32%)
> 1 year		41 (49%)	28 (37%)	45 (51%)
Previous episodes	1%			
0		19 (23%)	20 (26%)	22 (25%)
1		23 (28%)	12 (16%)	14 (16%)
2–3		17 (20%)	12 (16%)	15 (17%)
> 3		24 (29%)	32 (42%)	37 (42%)
Time since treatment initiated	2%			
< 2 weeks		18 (22%)	25 (33%)	12 (14%)
2–4 weeks		13 (16%)	10 (13%)	15 (17%)
> 4 weeks		52 (63%)	40 (53%)	60 (69%)
No. of health care visits for present LBP	2%			
1		16 (19%)	19 (25%)	15 (17%)
2–5		52 (63%)	45 (60%)	58 (67%)
6–10		10 (12%)	7 (9%)	12 (14%)
> 10		5 (6%)	4 (5%)	2 (2%)
Pain medication	1%			
None		38 (46%)	40 (56%)	39 (44%)
Over the counter		20 (24%)	15 (29%)	21 (24%)
Prescription		24 (29%)	18 (24%)	29 (33%)
START Back risk	6%			
Low	[any of 9 items missing]	41 (55%)	38 (51%)	40 (46%)
Medium		24 (32%)	22 (30%)	25 (29%)
High		10 (13%)	14 (19%)	22 (25%)
Oswestry Disability Index, mean (SD)	1% [≥ 4 of 10 items missing]	20.6 (11.2)	21.0 (12.3)	25.1 (11.9)
Back pain (0–10)	1%	5.0 (2.2)	5.7 (2.3)	5.0 (2.1)
Leg pain (0–10)	1%	2.5 (2.5)	3.0 (2.8)	3.0 (2.6)
Sick leave last 3 months*	1%			
0 days		41 (77%)	36 (77%)	36 (71%)
1–14 days		10 (19%)	7 (15%)	10 (20%)
> 15 days		2 (4%)	4 (9%)	5 (10%)
Illness perceptions, mean (SD)	2% [≥ 3 of 9 items missing]	39.9 (11.8)	41.1 (11.8)	42.1 (10.0)
Fear-avoidance beliefs, mean (SD)	5% [any of 4 items missing]	10.3 (5.9)	8.5 (5.9)	8.6 (5.9)
Mental wellbeing, mean (SD)	4% [any of 5 items missing]	70.7 (16.2)	72.5 (16.8)	71.9 (18.1)
Impaired social functioning	3%	11 (14%)	15 (20%)	24 (27%)
Back pain enablement	6% [any of 6 items missing]	40.4 (12.6)	41.5 (11.0)	39.5 (12.2)
Perceived physical fitness (0–40)	1%	18.7 (6.1)	20.4 (6.0)	17.7 (5.7)
Pain or restricted movement in forward bending	3%	49 (58%)	41 (58%)	60 (68%)
Seconds of trunk flexor endurance (0–120), mean (SD)	11%	52.6 (33.7)	55.5 (35.2)	49.1 (32.8)
Seconds of extensor endurance (0–180), mean (SD)	21%	76.8 (55.0)	88.6 (61.6)	71.9 (56.4)
Impaired straight leg raise test one or both sides	3%	28 (33%)	23 (32%)	27 (31%)

*Before-group* participants recruited prior to implementation of GLA:D Back, *after-group* participants recruited after the implementation

* Among those working

Active straight leg raise test was at baseline only impaired in approximately one third of the patients. The other performance tests showed a larger potential for improvements (Table 4).

### Reported treatments

Information/patient education either individually or in a group was part of the treatment in 36%, 33%, and 88% of patients in the before-group, after-group, and GLA:D Back groups respectively, while 80%, 69%, and 94% reported having received exercise therapy individually or in a group. Thus, some patients were registered in the GLA:D Back group by the clinician but did not report that they had received the intervention. Knowing that the complete intervention was offered in all clinics, this would be patients who either misunderstood the question on treatment received, who did not perceive GLA:D Back to patient education and exercises, or who were not compliant with the intervention. Ten patients in the before-group reported that they had participated in the GLA:D Back program prior to follow-up. Treatments were reported with similar frequencies in the before- and after-groups except for exercise therapy being less frequent in the after-group. Individual and group education and group exercises were more frequently reported in the GLA:D Back group than in the before- and after-groups, whereas individual exercise therapy, manual therapy, massage, and passive modalities were reported less frequent (Table 5).

**Table 5:** Treatments. Proportion of patients who reported they had received listed interventions as part of their treatment in the physiotherapy or chiropractic clinic

	Before-group	After-group	GLA:D Back group
Advice about daily activities	30 (59%)	26 (58%)	35 (51%)
Thorough information/education individually	12 (24%)	8 (24%)	28 (40%)
Thorough information/education in a group	13 (27%)	10 (22%)	62 (89%)
Exercise therapy individually	37 (71%)	29 (64%)	32 (48%)
Exercise therapy in a group	14 (29%)	9 (20%)	64 (91%)
Manual therapy	20 (43%)	24 (51%)	22 (33%)
Massage	24 (49%)	18 (42%)	12 (18%)
Passive modalities or needles (e.g. laser, ultrasound, acupuncture)	9 (20%)	9 (20%)	9 (14%)

*Before-group* participants recruited prior to implementation of GLA:D Back, *after-group* participants recruited after the implementation

### Patient outcomes

Generally, there were few missing values with a maximum of 6% missing on the START Back tool and the Back Pain Enablement Instrument (Table 4), which implied that the questionnaires and their administration were feasible to patients. Missing values were most common for the clinical tests of muscle endurance, i.e. 11% for trunk flexion and 21% for trunk extension (Table 4).

Improvements observed between baseline and the 4-month follow-up were generally small and slightly greater in the after-group as compared to the before-group, and for the GLA:D Back group when compared to both of the other groups (Table 6). However, it should be noted that this pilot study was not powered for statistical comparisons. Most patients reported high satisfaction with care, i.e. 76%, 78%, and 85% in the before-, after-, and GLA:D Back groups respectively.

**Table 6:** Observed (unadjusted) and marginal (adjusted) change scores from baseline to 4-month follow-up with 95% confidence intervals

		Before-group *n* = 63	After-group *n* = 59	GLA:D Back group *n* = 78
Oswestry Disability Index (0–100)	Unadjusted	1.8 (− 1.2; 4.8)	4.4 (1.7; 7.1)	6.5 (4.6; 8.4)
	Adjusted	2.4 (− 0.5; 5.3)	4.8 (1.9; 7.6)	5.7 (3.3; 8.1)
Back pain intensity (0–10)	Unadjusted	0.6 (−0.05; 1.3)	1.9 (1.2; 2.7)	1.2 (0.6; 1.7)
	Adjusted	0.8 (0.1; 1.5)	1.4 (0.7; 2.1)	1.3 (0.7; 1.9)
Leg pain intensity (0–10)	Unadjusted	0.3 (−0.4; 1.0)	0.9 (0.3; 1.6)	0.8 (0.2;1.4)
	Adjusted	0.7 (0.03; 1.4)	0.7 (−0.04; 1.4)	0.7 (0.1; 1.3)
Illness perceptions (0–80)	Unadjusted	2.7 (−1.2; 6.5)	3.6 (0.2; 7.1)	7.1 (4.3; 9.9)
	Adjusted	4.0 (0.07; 7.9)	2.7 (−1.2; 6.7)	6.7 (3.3; 10.0)
Fear-avoidance beliefs (0–24)	Unadjusted	1.3 (−0.2; 2.9)	2.3 (0.8; 3.7)	2.2 (1.0; 3.5)
	Adjusted	0.8 (−0.9; 2.5)	1.8 (0.2; 3.5)	2.2 (0.7; 3.6)
Mental well-being (0–100)	Unadjusted	4.7 (0.7; 8.8)	5.1 (0.2; 10.0)	7.3 (3.5; 11.1)
	Adjusted	4.0 (−0.2; 8.2)	6.1 (1.9; 10.4)	7.3 (3.8; 10.9)
Back pain enablement (0–60)	Unadjusted	2.9 (−0.4; 6.2)	2.8 (−0.4; 6.1)	5.9 (3.5; 8.4)
	Adjusted	2.3 (−0.6; 5.2)	2.4 (−0.5; 5.4)	5.5 (3.1; 7.9)
Perceived physical fitness (0–40)	Unadjusted	0.7 (−0.5; 1.8)	0.4 (−0.7; 1.5)	1.4 (0.4; 2.3)
	Adjusted	0.6 (−0.6; 1.7)	0.4 (−0.6; 1.5)	1.4 (0.4; 2.3)
Abdominal endurance, seconds	Unadjusted	9.0 (−0.5; 18.5) [*n* = 38]	27.8 (15.8; 39.8) [*n* = 17]	24.0 (16.8; 31.3) [*n* = 59]
	Adjusted	8.7 (−3.3; 20.7)	26.9 (11.9; 42.0)	22.3 (12.7; 31.9)
Extensor endurance, seconds	Unadjusted	13.3 (−1.6; 28.2) [*n* = 34]	41.9 (19.8; 65.1) [*n* = 16]	43.0 (27.9; 58.0) [*n* = 47]
	Adjusted	9.2 (−8.5; 26.8)	41.3 (18.2; 64.4)	46.5 (32.7; 60.4)
		Proportion at follow-up, %		
Impaired social functioning	Unadjusted	14%	14%	18%
	Adjusted	13%	15%	17%
High satisfaction	Unadjusted	76%	78%	85%
	Adjusted	81%	77%	85%
Pain or restricted movement in forward bending	Unadjusted	33% [*n* = 43]	32% [*n* = 25]	28% [*n* = 67]
	Adjusted	36%	31%	29%
Stopped pain medication*	Unadjusted	21%	18%	44%
	Adjusted	16%	17%	43%

Covariates in adjusted analyses: age, gender, education, START risk group, baseline value of covariate (when collected)

*Before-group* participants recruited prior to implementation of GLA:D Back, *after-group* participants recruited after the implementation

*Proportion not using pain medication among patient who used pain medication at baseline

### Modifications to the programme resulting from the pilot testing

Based on the pilot study, the clinician course was modified to emphasise and clarify the mandatory elements of GLA:D Back, emphasise the theory of the cognitive-behavioural approach more clearly, and to explain the rationale for the exercises more clearly.

In the clinical intervention, we modified the exercise programme to increase the span from the easiest to most difficult level. Also, the sit-to-stand test (number of repetitions from seated to standing within 30 s) was included in the modified programme as one physical test instead of the ASLR because the ASLR was negative in the majority of patients across groups and clinicians wanted a dynamic functional test.

### Recruitment in the before- and after-group

The main challenges observed for conducting a full-scale trial were related to patient recruitment for the before and after comparison groups. Some clinicians informed us that they would not be able to do the recruitment for instance because of usually having very few patients with the required profile or being too busy because of upcoming holidays. Clinicians who recruited patients were challenged by time and logistics and consecutive inclusion was not achieved:

testing… it takes longer than … this project has been costly… okay now that it is research and we did sign up,

it was right before the holidays and we had only one week, so we did not succeed in including any,

it [inclusion] was easier the second time … you could plan, make some timeslots.

Thus, recruitment rates in the before- and after-groups were considerably lower than expected (161 included when 600 expected), and it was very challenging to include consecutive LBP patients during pre-defined time periods in this setting.

The before- and after-groups were comparable on many parameters, but duration > 1 year, treatment for > 4 weeks, and higher education > 3 years were less frequent in the after-group than in the before-group, while it was the opposite for many previous episodes. The proportion of patients in the after-group that was enrolled in GLA:D Back was relatively low (10%) although the inclusion criteria for the before- and after-groups showed to match well with the characteristics of patients enrolled in GLA:D Back.

## Discussion

We tested the feasibility of implementing a standardised back pain intervention for people seeking care for persistent or recurrent back pain, GLA:D Back, in community-based clinics. The implementation was successful in the test clinics, and the intervention itself seems to work under daily routine conditions. However, recruitment of study participants by clinicians was cumbersome and the comparability of before- and after-groups is questionable.

Clinicians’ evaluations of the course were positive, and the clinical intervention was adopted by all test sites indicating good acceptance by clinicians. Scores on the PABS indicated an overall change in clinicians’ attitudes and beliefs in the intended direction towards a more behavioural orientation. The within-clinician change scores on the PABS demonstrated wide variation and a potential for substantial shifts in attitudes in some clinicians.

Patients enrolled in GLA:D Back mainly had persistent LBP (83% > 3 months) and had been in treatment for some time (69% > 4 weeks) indicating that clinicians mainly enrolled the target group of people with persistent or recurrent LBP that GLA:D Back was intended for.

Procedures for collecting patient outcomes were feasible, and the response rates and the completeness of patient-reported items were high. However, data from clinical tests were often missing at the end of treatment in the before- and after-groups where the tests were not part of the treatment programme.

Outcomes were generally better in the after- than in the before-group. With the small proportion (10%) receiving the new intervention, this may indicate a positive “spill over” effect of the clinician training to patients not participating in GLA:D Back. However, as discussed below, the comparison of these groups is not straightforward. Patient outcomes after participation in the GLA:D Back programme were as good or better than those in the before- and after-groups. The most pronounced improvements were captured on the Illness Perceptions Questionnaire and The Back Pain Enablement Instrument, which reflect targets of GLA:D Back, and the results for reducing use of pain medication were also promising. This study was however not powered for statistical comparisons, and these tendencies cannot be taken as evidence for effectiveness. Because the clinicians in the pilot study were voluntary participants with an interest in back pain and exercise therapy, we believe that the usual care delivered in these clinics represents a high standard for comparison. Therefore, benefits of GLA:D Back may be greater if a more diverse group of clinicians is trained to deliver GLA:D Back. Furthermore, some patients in the before-group participated in GLA:D Back prior to follow-up which may have reduced group differences. The results also showed promise for positive effects on the proportion of patients receiving recommended care. Importantly, even in these selected clinics, many patients reported not having received patient education prior to implementing the GLA:D Back programme, which suggests a need for structured programmes that can help clinicians deliver guideline-recommended treatments. A small proportion of patients enrolled in the GLA:D Back group did not report having received patient education and group-based exercises. This finding cannot be explained, but a more detailed registration of compliance will be incorporated in the GLA:D Back registry.

In contrast to the encouraging results about the intervention and implementation itself and about the outcomes we plan to use in GLA:D Back registry, we identified two major issues with respect to conducting a large-scale implementation-effectiveness study to evaluate a national implementation. Firstly, low recruitment rates combined with clinicians’ feedback indicate that consecutive recruitment of patients was not achieved even among this group of targeted motivated clinicians. Non-consecutive inclusion reduces generalisability, and selection of patients invited to the study may be affected by the implementation of GLA:D Back, which would bias result estimates. Secondly, even with consecutive inclusion, there is a considerable risk that before- and after-groups are non-comparable because implementation of GLA:D Back may affect the population of patients consulting the clinics shifting the population that is available for inclusion during the recruitment period. Thus, candidates for an intervention like GLA:D Back might have been enrolled at an earlier point, which was indicated by the larger proportion of patients in the after-group not having exercise therapy as part of their treatment.

Following consecutive patients from the first visit of a new pain episode will result in less selection bias, but only few of these patients may ever be candidates for GLA:D Back and may be enrolled much later thus diluting the observable effects. We also considered addressing the problem analytically, for example, by combining the after-group with a group of patients enrolled in GLA:D Back. However, we do not see a simple way to ensure that such a combined group does really match a before-group.

Consequently, we decided not to pursue a stepped wedge implementation-effectiveness study as part of the nationwide implementation. Particularly, it was challenging to test the effectiveness of the implementation intervention at the patient-level. The effectiveness of the GLA:D Back clinical intervention could be investigated in more traditional designs. We still plan to study the implementation process [13], and we plan to evaluate GLA:D Back in Denmark at national level by identifying relevant patient populations before and after implementation based on national registries. Moreover, we will use the GLA:D Back registry to closely monitor the included patients and their outcomes, as well as using the registry to investigate, for example in embedded trials, the room for improvement for the intervention and/or the training course [13].

## Conclusions

It was feasible to deliver the GLA:D Back clinician course as well as the clinical intervention in Danish primary care physiotherapy and chiropractic clinics. Because clinician satisfaction with the GLA:D Back course was high and effects on patient outcomes were at least as good as existing care in clinics considered to have a high standard of care, it is justified and relevant to continue implementation of GLA:D Back more widely. It was not deemed feasible to conduct an implementation-effectiveness trial such as a stepped-wedge trial as part of a nationwide implementation. Instead, outcomes of implementation will be monitored in the GLA:D Back clinical registry, which will inform the design of future effectiveness trials.

## Additional file

Additional file 1: Letter from The Regional Committees on Health Research Ethics for Southern Denmark. (PDF 60 kb)

## Abbreviations

ASLR: Active straight leg raise
B-IPQ: Brief Illness Perceptions Questionnaire
BPEI: Back Pain Enablement Instrument
GLA:D®: Good Life with osteoarthritis in Denmark. Trademark owned by University of Southern Denmark; only the abbreviation is used in relation to GLA:D Back
OPEN: Open Patient data Explorative Network
PABS: Pain Attitudes and Beliefs Scale
PCS: Practitioner Confidence Scale
REDCap: Research Electronic Data Capture
