# Supplementary material for: GLA:D® Back: group-based patient education integrated with exercises to support self-management of persistent back pain — feasibility of implementing standardised care by a course for clinicians
Source: Pilot Feasibility Stud. 2019 May 9;5:65. doi: 10.1186/s40814-019-0448-z (PMC6507160; doi:10.1186/s40814-019-0448-z)
Supplement: Supplementary file 1 — Letter from The Regional Committees on Health Research Ethics for Southern Denmark. (PDF 60 kb) [file 40814_2019_448_MOESM1_ESM.pdf]

Translation of letter from The Regional Committees on Health Research Ethics for Southern Denmark

Dear Alice Kongsted,

The Regional Committees on Health Research Ethics for Southern Denmark have received the request below regarding notification of your study, which has received Case Number S-20172000-93.

Based on the information available, the Committee has decided that the project must not be notified to scientific ethical committee system, cf. § 14 1 of the Act on Scientific Ethics of health science research projects.

The decision emphasizes that this seems to be a questionnaire survey that falls outside the scope of the committee's definition of a health science research project. The project may need to be notified to the Data Protection Agency or reported to an existing institutional authorisation and the project may require permission from the National Board of Health, if the project entails disclosure of patient record information - see more here: http://stps.dk/da/sundhedsprofessionelle-ogyndigheder/patientjournaloplysninger

The case has been dealt with by the chair of Committee 2, Professor, chief doctor, Dr. Jens Michael Hertz. The committee's decision may, cf. § 26, subsection 1, be submitted to the National Science Ethics Committee (NVK), not later than 30 days after the decision has been received. NVK may, in order to ensure the safety of the subjects' rights, treat elements of the project that are not covered by the complaint itself. The complaint must be submitted electronically and using digital signature and encryption if the protocol contains confidential information. This can be done at: dketik@dketik.dk The complaint must be justified and include the decision of the regional scientific committee as well as the files which the decision by the Regional Scientific Committee was based on. NB: No changes may be made to the documents that have been submitted to the committee, or it will otherwise be returned to the committee.

Sincerely,

Christina Fly

Christina Sølvsten Fly

Administrative Coordinator

Quality and Research, the Scientific Ethics Committees for the Region of Southern Denmark
